# Analysis of the Delta Variant B.1.617.2 COVID-19

**DOI:** 10.3390/clinpract11040093

**Published:** 2021-10-21

**Authors:** Shayan Shiehzadegan, Nazanin Alaghemand, Michael Fox, Vishwanath Venketaraman

**Affiliations:** 1 Department of Basic Medical Sciences, College of Osteopathic Medicine of the Pacific, Western University of Health Sciences, 309 E 2nd St, Pomona, CA 91766, USA; shayan.shiehzadegan@westernu.edu (S.S.); michael.fox@westernu.edu (M.F.); 2 Department of Biology, University of California Irvine, Irvine, CA 92697, USA; nalaghem@uci.edu

**Keywords:** COVID-19, delta variant, B.1.617.2

## Abstract

With the delta variant of COVID-19, known as B.1.617.2, quickly ramping up infections around the world, we need to understand what makes this variant more contagious. One study has reported that the delta variant is 60% more transmissible than the alpha variant. As of August 2021, the delta variant has quickly become the dominant strain. Despite countries like the US, where most of the population is vaccinated, COVID-19 has made a resurgence in infections. Collectively, as a country, we ask: is it more deadly? What makes it more “contagious” or “transmissible”? This review article delves into the information we already know about the delta variant and how it compares with the other SARS-CoV-2 variants. The current vaccine companies like AstraZeneca, Pfizer/BioNTech, and Moderna have reported that their vaccines can provide protection against this variant but with a slightly reduced efficacy. In this article, we do a comprehensive review and summary of the delta B.1.617.2 variant and what makes it more contagious.

## 1. Introduction

The recently discovered novel virus, Severe Acute Respiratory Syndrome Coronavirus 2 (SARS-CoV-2), was initially found in late 2019 in Wuhan, China [1]. Coronavirus disease 19 (COVID-19) is a zoonotic disease, and it is widely believed that SARS-CoV-2 stemmed from animals like bats, snakes, and pangolins found in the market located in Wuhan [1]. According to Worldometers, SARS-CoV-2 has infected over 188 million people, and around 4 million deaths have resulted from this virus worldwide [2].

In addition to the millions of lives lost from COVID-19, the pandemic continues to have a tremendous impact on both the mental and physical health of various individuals. Healthcare workers are not the only group that have undergone excessive stress due to this pandemic; the general population has illustrated psychiatric symptoms as well [3]. The growing concern among the people about the possibility of acquiring SARS-CoV-2 infection has greatly increased depression, stress, confusion, and anxiety in the general population [4]. One study showed that SARS-CoV-2 infection had significantly decreased the amount of physical activity per week in Italy [5]. Physical inactivity can lead to significant increase in the rates of cardiac-related deaths [6]. The impacts of COVID-19 are significant on humans’ overall health; thus, taking a look at this delta variant that is causing a recent surge of COVID-19 cases in the US, Europe, and other parts of the world is crucial to the public health.

There are currently four variants that the CDC is monitoring. The first SARS-CoV-2 virus is B.1.1.7. This is called the alpha variant, and it was initially detected in the United Kingdom [7]. B.1.351 is the beta variant that was first detected in South Africa. P.1 is the gamma variant that was first detected in Brazilian travelers. The new variant of SARS-CoV-2 known as the delta variant was originally found in India in December 2020 [8]. The delta variant has spread over 60 countries very fast because of its capability to invade the host’s immune system compared to the original strain [6]. Over 26% of the Indian population was infected with the delta variant in a three-month period. The high rate of transmission is most likely due to the immune evasive property of the delta strain. Additionally, during the second pandemic wave, there was a loss in population immunity, which was a result of the delta variant [8]. In the US, the infection rates continue to increase despite vaccination efforts and pandemic restrictions [9].

## 2. Pathogenesis of B.1.617.2

**Figure 1:**
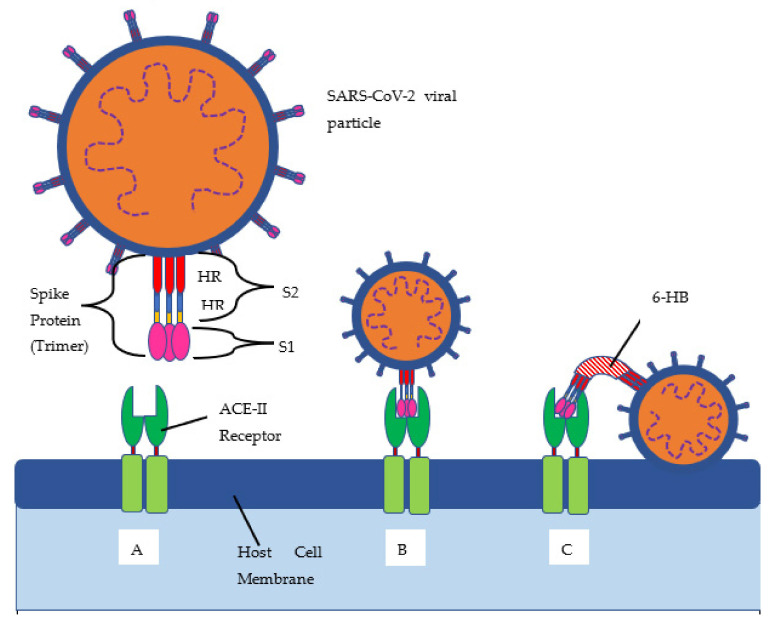
SARS-CoV-2 structure and binding schematic. (**A**) Native resting state of the ACE-II receptor on the host cell surface. (**B**) Spike protein binding to the ACE-II receptor. (**C**) Viral fusion with the host cell membrane. Host cell proteases cleave the spike protein into S1 and S2 subunits. S1 is responsible for binding to ACE-II, while S2 is responsible for viral fusion and entry. Upon S1 binding ACE-II, the HR 1 and 2 subunits form the 6-helix bundle (6-HB) in order to bring the viral particle close to the host cell to allow for fusion and entry [11].

**Figure 2:**
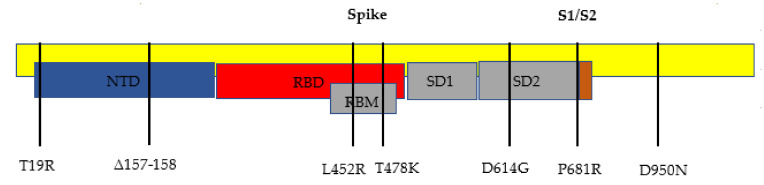
Representation of the spike protein mutations found in the B.1.6172 variant. NTD = N-terminal domain, RBD = receptor-binding domain, RBM = receptor-binding motif, SD1 = subdomain 1, and SD2 = subdomain 2.

The delta variant of SARS-CoV-2, B.1.617.2, has 23 mutations compared to the first identified COVID-19 strain (alpha strain) [10]. Twelve of those mutations are in the spike protein (see Figure 1 and Figure 2, and Table 1 below). The spike protein allows for the attachment of host cells to enable entry into the cells. The spike protein is also the protein that is targeted by the immune system for eradication of the virus. Once the spike protein is recognized as foreign by the immune system, B cells produce antibodies to attach to this spike protein for eradication. The spike protein is composed of two subunits, called S1 and S2. S1 binds to the ACE2 receptor, and S2 aids with the fusion and integration of the virus to the host cell [11]. The more the spike proteins mutate, the harder it is for the immune system to identify them and for the antibodies to attach for the subsequent eradication of the virus. This new spike protein evading the immune system allows for a better attachment to human cells, thus infecting them more effectively. 

## 3. Delta Variant Mutations

The most notable gene mutations that are suspected to allow the delta variant to be the most transmissible variant yet are the mutations found in the spike proteins. The spike gene mutations in this B.1.617.2 variant are T19R, L452R, T478K, D614G, P681R, and d960N, with deletions at positions 157 and 158.

Most notable are the L452R and P681R spike protein mutations. The L452R mutation substitutes an arginine for a leucine at position 452. One study suggests that this allows for the spike protein to attach to the ACE2 receptor with a higher affinity. The ACE2 receptor is a receptor found in the human host in many cells of the body that allows the spike protein of SARS-CoV-2 to bind to this receptor. This may help evade vaccine-stimulated antibodies to bind to the spike protein, because the ACE2 receptor is bound with the spike protein with a higher affinity [13]. Yet, other studies have shown that the L452R mutation can allow the delta variant to evade being attacked by CD8 T cells, which are the cells that eradicate the virus [14].

The other notable mutation of the B.1.617.2 variant is the substitution P681R. The arginine substitutes the proline at position 681, and this mutation helps cleave the precursor spike protein to the activated forms of the spike protein called S1 and S2 [15,16]. This would allow for superior fusion and integration of the virus to the host cell as compared to variants without this mutation.

## 4. Epidemiology of the Delta Variant

According to the CDC, the delta variant spreads twice as easily as the alpha variant [17]. An infection and death rate profile from the WHO Coronavirus (COVID-19) Dashboard shows the various waves, with the second wave starting around February of 2021 and consisting of a higher percentage of the delta variant than the previous spike [18]. The American Society for Microbiology reports that this newer strain accounts for 83% of cases in the US and 90% in the UK. They even report a 40–60% increase in transmissibility compared to the alpha variant, which itself was twice as contagious as the original strain from Wuhan [19]. It is even apparent on a qualitative level that the infection and death rates spiked much more rapidly despite occurring in a much shorter timeframe. The peak cases in the first wave were 5,001,049 vs. 5,703,208 in the second; the peak death rate in the first wave was 101,084 vs. 96,684 in the second; and the first wave gradually increased over March, until it rapidly spiked from about October 2020 to February 2021, when the second wave rapidly spiked from February 2021 to about June [18]. On a quantitative level, the delta variant has been shown to have a 108% increase in hospitalization risk, 235% increase in ICU admission, and 133% higher chance of death than the original variant [20].

One study in Scotland showed that the delta variant led to double the risk for hospitalization as compared to the alpha variant. The study also suggested that the delta variant was more common in their younger and affluent populations [21]. Another study showed that the delta variant has been spread rapidly in UK primary and secondary schools [22]. Even though the UK is a country with a high vaccination rate, it is of concern to see an outbreak of the delta variant in primary and secondary schools because of students in the US starting school in the fall season. Since the outbreaks have been mainly of school-aged children, and since these school-aged children are the last to get the vaccine, this suggests that adults who have gotten the vaccine are more protected than children who have not gotten the vaccine. However, more data is needed to understand why school-aged children are acquiring the delta variant—is it their unvaccinated status alone, or is it their unvaccinated status combined with a less fully developed immune system that is common in young children?

High rates of transmission in the delta variant can lead to high rates of mutations and the emerging of new strains. Due to the appearance of new variants of the coronavirus, Moderna and Pfizer may likely need to produce booster doses for their vaccines.

As for vaccinated individuals getting breakthrough COVID-19 infections, a study in the UK found that vaccinated individuals have similar protection against the delta variant as they do with the alpha variant. Bernal et al. found that a single dose of either the BNT162b2 or ChAdOx1 nCoV-19 will have similar efficacies of 30.7% against the delta vs. 48.7% against the alpha. Two doses of BNT162b2 were found to be 93.7% effective against the alpha vs. 88% effective against the delta. ChAdOx1 nCoV-19 was found to be 74.5% effective against the alpha and 67% effective against the delta [23]. Another study showed that the Pfizer/BioNTech vaccine showed up to 88% protection, although not as effectively as against the alpha variant [23]. Due to a decrease in effectiveness of the coronavirus vaccines to new variants of SARS-CoV-2, it is expected that the number of deaths from coronavirus will increase in the next twelve months [24]. Thus, it is expected that the reduction in effectiveness of the COVID-19 vaccines will lead to a surge of a new pandemic. However, various studies still support that vaccination will still decrease the chances of morbidity and mortality from the new strain.

Besides a diminished efficacy in the ability of the vaccines to prevent infection, properties of infected individuals create further problems with containing the spread. One study demonstrated that fully vaccinated individuals with breakthrough infections have a similar viral load as unvaccinated patients [25]. Further complicating the issue is a finding that, among infected patients, non-hospitalized ones can even have a higher viral load than hospitalized patients [26]. Since much of COVID-19 treatment and quarantining is based on symptomatology and not necessarily viral load, infected individuals can continue to readily spread the delta variant despite the current public health efforts.

## 5. Common Symptoms of the Delta Variant

Some common symptoms for the delta variant are fever, cough, shortness of breath, vomiting, diarrhea, sore throat, and headache [20,27]. Other symptoms include: myalgias, loss of taste, loss of smell, fatigue, and rhinorrhea [28]. Currently, studies indicate that the symptoms of the delta variant and alpha variant are similar, but patients with the delta variant become more rapidly ill and grow higher viral loads in the respiratory tract. Examinations in the UK have shown the delta variant to uniquely cause auditory impairment and gangrene from worse blood clots while less commonly causing cough and loss of the sense of smell [19]. More studies and case reports are needed to document if the delta variant truly causes different symptoms from the alpha variant in order to clarify the conflicting reports.

## 6. Discussion

The B.1.617.2 variant is quickly becoming the common variant of the COVID-19 pandemic. The areas in the US that are showing the cases of the delta variant are in the under-vaccinated populations. However, the cases in all 50 states are increasing, but this can be a combination of the delta variant’s easier transmissibility, as well as Americans using less masks and social distancing. More strains can emerge from the delta variant as more people get infected. Due to the seriousness of this new delta variant, the Centers for Disease Control and Prevention has now recommended that even vaccinated individuals should wear masks indoors in populations where transmission rates of COVID-19 are high. This happened after the daily COVID-19 cases have increased by four-fold since the beginning of July 2021.

We call for a better look at the treatment of patients infected with the delta variant. Since the symptoms are different than the alpha variant, we need more research looking at what the best way is to help these patients who are infected with the delta variant. For example, since steroids have been used in the alpha variant, are steroids still appropriate to be used in patients infected with the delta variant, even though they do not commonly present with difficulty breathing? A lot of information is known about the other variants of the SARS-CoV-2 virus, but still, not much is known about the delta variant of this virus. As more people get infected with the delta variant, more information will become available, but one way to continue combatting this virus is to continue social distancing, use face masks, use proper hygiene, and become fully vaccinated.

## 7. Conclusions

More studies need to be done to fully assess the delta variant, but in this comprehensive review, we investigated what makes this delta variant more contagious, the associated symptoms, and the social impacts of this virus. It seems that the variant’s higher infectivity is a combination of key mutations giving the spike protein higher affinity binding of ACE-II, decreased efficacy of vaccines against it, and higher viral loads in infected individuals. More research is needed to clarify the unique symptoms of this strain, as well as infection profiles. Hopefully, newfound knowledge will help guide treatments and prevention through vaccination strategies and other public policies.

## Figures and Tables

**Table 1:** Characteristic mutations of B.1.617.2 [12]. S = spike protein gene, N = nucleocapsid gene, ORF = open reading frames gene, and M = membrane protein gene.

Gene	Amino Acid
S	T19R
S	del157/158
S	L452R
S	T478K
S	D614G
S	P681R
S	D950N
ORF1a	A1306S
ORF1a	P2046L
ORF1a	P2287S
ORF1a	V2930L
ORF1a	T3255I
ORF1a	T3646A
ORF1b	P314L
ORF1b	G662S
ORF1b	P1000L
ORF1b	A1918V
ORF3a	S26L
M	I82T
ORF7a	V82A
ORF7a	T120I
ORF7b	T40I
ORF8	del119/120
N	D63G
N	R203M
N	D377Y

## Data Availability

The gene mutations of variant B.1.617.2: https://outbreak.info/situation-reports?pango=B.1.617.2 (accessed on 9 October 2021).

## Abbreviations

ACE 2 = angiotensin-converting enzyme, B.1.1.7 = alpha variant COVID-19, B.1.351 = beta variant COVID-19, B.1.617.2 = delta variant COVID-19, CD8 = Cluster of Differentiation 8, CDC = Centers for Disease Control and Prevention, COVID-19 = Coronavirus 19, ICU = intensive care unit, NTD = N-terminal domain, P.1 = gamma variant COVID-19, RBD = receptor-binding domain, RBM = receptor-binding motif, S1/2 = spike protein subunit ½, SARS-CoV-2 = Severe Acute Respiratory Syndrome Coronavirus 2, SD1 = subdomain 1, SD2 = subdomain 2, and WHO = World Health Organization.

## References

[1] Ji W., Wang W., Zhao X., Zai J., Li X. (2020). Cross-Species Transmission of the Newly Identified Coronavirus 2019-NCoV. J. Med. Virol..

[2] Worldometer (2021). Coronavirus Cases. www.worldometers.info/coronavirus/#countries.

[3] Pappa S., Ntella V., Giannakas T., Giannakoulis V.G., Papoutsi E., Katsaounou P. (2020). Prevalence of Depression, Anxiety, and Insomnia among Healthcare Workers during the COVID-19 Pandemic: A Systematic Review and Meta-Analysis. Brain Behav. Immunity.

[4] Shigemura J., Ursano R.J., Morganstein J., Kurosawa M., Benedek D.M. (2020). Public responses to the novel 2019 coronavirus (2019-nCoV) in Japan: Mental health consequences and target populations. Psychiatry Clin. Neurosci..

[5] Maugeri G., Castrogiovanni P., Battaglia G., Pippi R., D’Agata V., Palma A., Di Rosa M., Usumeci G. (2020). The Impact of Physical Activity on Psychological Health during COVID-19 Pandemic in Italy. Heliyon.

[6] Doukky R., Mangla A., Ibrahim Z., Poulin M.F., Avery E., Collado F.M., Kaplan J., Powell L.H. (2016). Impact of Physical Inactivity on Mortality in Patients With Heart Failure. Am. J. Cardiol..

[7] Callaway E. (2021). Delta Coronavirus Variant: Scientists Brace for Impact. Nature.

[8] Yang W., Jeffrey S. (2021). COVID-19 Pandemic Dynamics in India and Impact of the SARS-CoV-2 Delta (B.1.617.2) Variant. MedRxiv.

[9] Irfan U. (2021). How the Delta Variant Is Altering the Course of the Pandemic. Vox Media. www.vox.com/22547537/delta-coronavirus-variant-COVID-19-vaccines-masks-lockdown.

[10] Hodcroft E.B. SARS-CoV-2 Mutations and Variants of Interest. CoVariants. https://covariants.org/.

[11] Huang Y., Yang C., Xu X.F., Xu W., Liu S.W. (2020). Structural and functional properties of SARS-CoV-2 spike protein: Potential antivirus drug development for COVID-19. Acta Pharmacol. Sin..

[12] Latif A.A., Mullen J.L., Alkuzweny M., Tsueng G., Cano M., Haag E. Center for Viral Systems Biology. 1 July 2021. B.1.617.2 Lineage Report. https://outbreak.info/situation-reports?pango=B.1.617.2.

[13] Starr T.N., Greaney A.J., Dingens A.S., Bloom J.D. (2021). Complete Map of SARS-CoV-2 RBD Mutations That Escape the Monoclonal Antibody LY-CoV555 and Its Cocktail with LY-CoV016. Cell Rep. Med..

[14] Koshy J. (2021). Coronavirus|Indian ‘Double Mutant’ Strain Named B.1.617. The Hindu. https://www.thehindu.com/news/national/indian-double-mutant-strain-named-b1617/article34274663.ece.

[15] Haseltine W. (2021). An Indian SARS-CoV-2 Variant Lands in California. More Danger Ahead?. Forbes.

[16] Bertram S., Dijkman R., Habjan M., Heurich A., Gierer S., Glowacka I., Welsch K., Winkler M., Schneider H., Hofmann-Winkler H. (2013). TMPRSS2 Activates the Human Coronavirus 229E for Cathepsin-Independent Host Cell Entry and Is Expressed in Viral Target Cells in the Respiratory Epithelium. J. Virol..

[17] CDC (2021). Delta Variant: What We Know about the Science. COVID-19. https://www.cdc.gov/coronavirus/2019-ncov/variants/delta-variant.html.

[18] WHO (2021). WHO Coronavirus (COVID-19) Dashboard. https://covid19.who.int/.

[19] Hagen A. (2021). How Dangerous Is the Delta Variant (B.1.617.2)? American Society for Microbiology. https://asm.org/Articles/2021/July/How-Dangerous-is-the-Delta-Variant-B-1-617-2.

[20] Lang K. (2021). Delta Variant has 235% Higher Risk of ICU Admission than Original Virus. Medical News Today.

[21] Sheikh A., McMenamin J., Taylor B., Robertson C. (2021). SARS-CoV-2 Delta VOC in Scotland: Demographics, risk of hospital admission, and vaccine effectiveness. Lancet.

[22] Torjesen I. (2021). COVID-19: Delta variant is now UK’s most dominant strain and spreading through schools. BMJ.

[23] Bernal J.L., Andrews N., Gower C., Gallagher E., Simmons R., Thelwall S., Stowe J., Tessier E., Groves N., Dabrera G. (2021). Effectiveness of COVID-19 Vaccines against the B.1.617.2 (Delta) Variant. N. Engl. J. Med..

[24] Li R., Li Y., Zou Z., Liu Y., Li X., Zhuang G., Shen M., Zhang L. (2021). Projecting the Impact of SARS-CoV-2 Variants on the COVID-19 Epidemic and Social Restoration in the United States: A Mathematical Modelling Study. MedRxiv.

[25] Tucker R. (2021). COVID Delta Variant Viral Load Similar in Vaccinated and Unvaccinated. Hospital Healthcare Europe. https://hospitalhealthcare.com/COVID-19/COVID-delta-variant-viral-load-similar-in-vaccinated-and-unvaccinated/.

[26] Argyropoulos K.V., Serrano A., Hu J., Black M., Feng X., Shen G., Call M., Kim M.J., Lytle A., Belovarac B. (2020). Association of Initial Viral Load in Severe Acute Respiratory Syndrome Coronavirus 2 (SARS-CoV-2) Patients with Outcome and Symptoms. Am. J. Pathol..

[27] UC Davis Health (2021). Delta Variant: 8 Things You Should Know. Coronavirus.

[28] Tellez D., Dayal S., Phan P., Mawley A., Shah K., Consunji G., Tellez C., Ruiz K., Sabnis R., Dayal S. (2021). Analysis of COVID-19 on Diagnosis, Vaccine, Treatment, and Pathogenesis with Clinical Scenarios. Clin. Pract..

